# Population analysis of heavy metal and biocide resistance genes in *Salmonella enterica* from human clinical cases in New Hampshire, United States

**DOI:** 10.3389/fmicb.2022.983083

**Published:** 2022-10-19

**Authors:** Stephanie S. R. Souza, Madison R. Turcotte, Jinfeng Li, Xinglu Zhang, Kristin L. Wolfe, Fengxiang Gao, Christopher S. Benton, Cheryl P. Andam

**Affiliations:** ^1^Department of Biological Sciences, University at Albany, State University of New York, Albany, NY, United States; ^2^New Hampshire Department of Health and Human Services, Concord, NH, United States

**Keywords:** *Salmonella enterica*, population genomics, heavy metal resistance, biocide resistance, antimicrobial resistance

## Abstract

Microbes frequently encounter heavy metals and other toxic compounds generated from natural biogeochemical processes and anthropogenic activities. Here, we analyzed the prevalence and association of genes conferring resistance to heavy metals, biocides, and antimicrobial compounds in 394 genome sequences of clinical human-derived *S. enterica* from New Hampshire, USA. The most prevalent was the gold operon (*gesABC-golTSB*), which was present in 99.2% of the genomes. In contrast, the other five heavy metal operons (arsenic, copper, mercury, silver, tellurite) were present in 0.76% (3/394)–5.58% (22/394) of the total population. The heavy metal operons and three biocide resistance genes were differentially distributed across 15 sequence types (STs) and 16 serotypes. The number of heavy metal operons and biocide resistance genes per genome was significantly associated with high number of antimicrobial resistance (AMR) genes per genome. Notable is the mercury operon which exhibited significant association with genes conferring resistance to aminoglycosides, cephalosporins, diaminopyrimidine, sulfonamide, and fosfomycin. The mercury operon was co-located with the AMR genes *aac(3)-IV*, *ant(3”)-IIa*, *aph(3’)-Ia*, and *aph(4)-Ia, CTX-M-65, dfrA14*, *sul1*, and *fosA3* genes within the same plasmid types. Lastly, we found evidence for negative selection of individual genes of each heavy metal operon and the biocide resistance genes (dN/dS < 1). Our study highlights the need for continued surveillance of *S. enterica* serotypes that carry those genes that confer resistance to heavy metals and biocides that are often associated with mobile AMR genes. The selective pressures imposed by heavy metals and biocides on *S. enterica* may contribute to the co-selection and spread of AMR in human infections.

## Introduction

Foodborne illnesses caused by the gram negative bacterium *Salmonella enterica* is a global public health concern (Chlebicz and Śliżewska, 2018; Popa and Popa, 2021), causing considerable morbidity, mortality, and economic burden (Tauxe et al., 2010). The main reservoir of *S. enterica* is the intestines of humans and mammals, but the organism has also been identified in cold-blooded reptiles and insects (Mermin et al., 2004; Soto-Arias et al., 2014). The main source of infection in humans are contaminated food products and water (Chlebicz and Śliżewska, 2018). Based on the disease caused, *S. enterica* can be divided into typhoidal and non-typhoidal *Salmonella* (NTS). The typhoidal serotypes (Typhi and Paratyphi) often cause life-threatening enteric fever, while NTS are frequently associated with diarrhea or self-limiting gastroenteritis in humans (Cheng et al., 2019).

Multidrug resistant (MDR) *S. enterica*, defined as strains exhibiting resistance to the first line antimicrobials ampicillin, chloramphenicol, and co-trimoxazole, have been documented worldwide (Phoon et al., 2015; Shrestha et al., 2016; Mutai et al., 2018; Browne et al., 2020) and represents a serious challenge in the treatment and control of *S. enterica* infections. In the United States, the Centers for Disease Control and Prevention [CDC] (2013) estimates 212,500 infections caused by drug-resistant NTS and 4,100 infections caused by drug-resistant *Salmonella* Typhi (Centers for Disease Control and Prevention [CDC], 2019). In lower-middle income countries, the percentage of drug-resistant *Salmonella* can reach up to 70% of total isolates (Tack et al., 2020). The high prevalence of MDR strains also contributes to an increase in mortality rates of *Salmonella* infections in high-income countries (Parisi et al., 2018).

Understanding the drivers that contribute to the spread of drug-resistant *S. enterica* is essential to responsive decision-making related to infection control and public health system decision policies. In this context, the presence of compounds containing heavy metals, metalloids, biocides and other toxic compounds has been associated with the maintenance and proliferation of antimicrobial resistance (AMR) (Baker-Austin et al., 2006; Dickinson et al., 2019). Microbes frequently encounter metals in their habitats mainly because heavy metals are released as part of natural planetary biogeochemical activities. Anthropogenic activities also generate high levels of these compounds to which bacteria are exposed to (Vareda et al., 2019). Heavy metals such as mercury, copper, silver and arsenic have a long history of human usage in medicine and agriculture, including as biocides, feed additives, animal growth promoters and antimicrobials (Hobman and Crossman, 2015; Rensing et al., 2018). Other rare metals such as gold have been used recently in medical diagnostics or imaging and as anti-arthritis and cancer therapeutics (Hobman and Crossman, 2015). Metals are also present in many consumer products, from clothing to computer keyboards, and in industrial emissions and fossil fuels combustion (Vareda et al., 2019; Hubeny et al., 2021).

Heavy metals can accumulate throughout all levels of the food chain, including soil and water sources (Kumar et al., 2019). The presence of heavy metals and metalloids can have toxic effects on the resident microbial communities (Shuaib et al., 2021), affecting the biomass (Yuangen et al., 2004), diversity (Berg et al., 2012), and metabolic activity (Li et al., 2019). To overcome such stress, bacteria can evolve a variety of resistance mechanisms to heavy metals and other toxic compounds, mediated by chromosomal mutations or the acquisition of exogenous resistance genes carried on mobile genetic elements (Chandrangsu et al., 2017). Genes related to metals and metalloids are often carried on the same mobile elements where AMR genes, virulence factors and other accessory genes are co-localized (Dziewit et al., 2015; Pal et al., 2015; Bukowski et al., 2019).

In this study, we sought to investigate (1) the phylogenetic distribution of heavy metal and biocide resistance genes in clinical *S. enterica* isolates; (2) their co-occurrence with AMR genes; and (3) the impact of selection on these genes in genome sequences of 394 clinical human-derived *S. enterica* from the state of New Hampshire, USA. We detected the differential distribution of six heavy metal operons (arsenic, copper, gold, mercury, silver, tellurite) and three biocide resistance genes (*qacEdelta1*, *qacL*, and *sugE1*) across phylogenetically diverse lineages. Our study highlights the need for continued surveillance of genotypes that carry those genes that confer resistance to heavy metals, biocides, disinfectants and other toxic compounds and that are often associated with mobile AMR genes. The selective pressures imposed by heavy metals and biocides on *S. enterica* may contribute to the selection and spread of AMR in human infections.

## Materials and methods

### Genomic data set

Our data set consisted of 394 *S. enterica* genomes that have been fully characterized in a previous study from our group (Turcotte et al., 2022). The genomes were recovered from bacterial isolates received by the Public Health Laboratories of the New Hampshire Department of Health and Human Services (DHHS) from healthcare providers from January 2017 to July 2020. The isolates were recovered from patients who were diagnosed with *Salmonella* infections by health care providers across the state of New Hampshire. There were 15 isolates that were obtained from patients who came from neighboring states, but who were diagnosed in New Hampshire. A total of 44 isolates were collected in 2017, 158 isolates in 2018, 156 isolates in 2019, and 36 isolates in 2020. Isolates from 2018 to 2020 represented all *S. enterica* isolates received by the DHHS. In 2017, only those *S. enterica* isolates that were specifically requested by state epidemiologists at the Bureau of Infectious Disease Control (BIDC) or the CDC were sequenced. No identifiable information is associated with the isolates. These isolates were part of a nationwide surveillance program PulseNet (Tolar et al., 2019) of CDC, which links foodborne illness cases through a national laboratory network to detect disease outbreaks.

DNA extraction, whole genome sequencing and initial genome characterization were carried out as previously described (Turcotte et al., 2022). Following the protocol provided by PulseNet,^1^ DNA extraction was carried out using the DNeasy Blood and Tissue Kit (Qiagen, Valencia CA). Genomic DNA for each isolate was used to construct sequencing libraries using the Nextera XT DNA Library Preparation Kit (Illumina, Inc., San Diego, CA). Multiplexed DNA libraries were sequenced on the Illumina MiSeq platform to produce paired end reads of 250 bp in length. Genome assembly was carried out using Shovill v.1.1.0^2^ and annotation using Prokka v.1.14.5 (Seemann, 2014). Genome quality was assessed using QUAST v.5.0.2 (Gurevich et al., 2013) and CheckM v.1.1.3 (Parks et al., 2015). Sequence type (ST) of each genome was determined using MLST v.2.19.0.^3^ The k-mer-based algorithm SeqSero2 was used to predict the serotype based on the sequences of the O and H antigens (Zhang et al., 2019). AMR genes were identified using ABRicate v.1.0.0^4^ applying threshold value of > 95% sequence identity and > 95% sequence coverage to known resistance genes deposited in the Comprehensive Antibiotic Resistance Database (Alcock et al., 2020). Phylogenetic relationships were reconstructed using a core-SNP based maximum likelihood tree using SNP-site v.2.5.1 and RAxML (Stamatakis, 2014; Page et al., 2015, 2016). We used the alignment of 225,784 SNPs from 3,265 core genes to build the phylogeny. Population clustering analysis was implemented employing the R-implemented Bayesian hierarchical clustering algorithm Bayesian Analysis of Population Structure (RhierBAPS) (Tonkin-Hill et al., 2018). Accession numbers, sources, and genomic features of each isolate are listed in Supplementary Table 1.

### *In silico* detection of heavy metal, biocide, and antimicrobial resistance genes

We used AMRFinderPlus v. 3.10 (Feldgarden et al., 2021) to screen all genomes for the presence of heavy metal and biocide resistance genes. For the six heavy metal elements found, we only considered complete operons as follows: for arsenic operon—*arsR* (As(III)-sensing metalloregulatory transcriptional repressor), *arsB* (arsenite efflux transporter ATPase subunit), *arsC* (arsenate reductase)*;* for copper operon—*pcoA* (multicopper oxidase PcoA), *pcoB* (copper-binding protein PcoB), *pcoC* (copper resistance system metallochaperone PcoC), *pcoD* (copper resistance inner membrane protein PcoD), *pcoR* (copper response regulator transcription factor PcoR), *pcoS* (copper resistance membrane spanning protein PcoS), and *pcoE* (copper resistance system metallochaperone PcoE)*;* for gold operon—*gesA* (multidrug efflux RND transporter periplasmic adaptor subunit MdsA), *gesB* (multidrug efflux RND transporter periplasmic adaptor subunit MdsB), *gesC* (multidrug efflux RND transporter periplasmic adaptor subunit MdsC), *golT* (gold/copper-translocating P-type ATPase GolT) and *golS* (Au(I) sensor transcriptional regulator GolS)*;* for mercury operon—*merR* (mercuric resistance transcriptional regulator), *merT* (mercuric transporter), *merP* (mercuric resistance system periplasmic binding protein), and *merA* (mercury (II) reductase)*;* for silver operon—*silE* (silver-binding protein SilE), *silS* (copper/silver sensor histidine kinase SilS), *silR* (copper/silver response regulator transcription factor SilR), *silC* (Cu(+)/Ag(+) efflux RND transporter outer membrane channel SilC), *silF* (Cu(+)/Ag(+) efflux RND transporter periplasmic metallochaperone SilF), *silB* (Cu(+)/Ag(+) efflux RND transporter periplasmic adaptor subunit SilB) and *silA* (Cu(+)/Ag(+) efflux RND transporter permease subunit SilA); and for tellurite operon—*terB* (tellurium resistance membrane protein TerB), *terC* (tellurium resistance membrane protein TerC), *terD* (tellurium resistance membrane protein TerD) and *terE* (tellurium resistance cAMP binding protein TerE).

For those genomes in which the genes *golT* and *golS* (part of the gold resistance operon) were detected, we created a custom database in ABRicate v.1.0.0 to detect the rest of the genes related to the operon (*gesABC* and *golB*) (Pontel et al., 2007). We also included two other genes associated with quaternary ammonium compound resistance (*sugE1*/*sugE2*) in the custom database. We applied a threshold value of > 75% sequence identity and > 85% sequence coverage to expand our search space of heavy metal and biocide resistance genes. The accession numbers for the genes used to construct the custom database are listed in Supplementary Table 2.

### Plasmid reconstruction

We used the mob-recon tool from the MOB-suite software (Robertson and Nash, 2018; Robertson et al., 2020) to determine whether the heavy metal operons, biocide resistance genes, and AMR genes were associated with plasmid elements. We used the assembled genomes as input and the basic mode of the mob-recon tool. The fasta files generated from the bacterial chromosomes and the predicted plasmids were both analyzed to confirm the localization of heavy metal operons, biocides, and AMR genes using AMRFinderPlus and ABRicate using the same settings as described above.

### Selection analysis

To investigate the extent of selective pressure operating on heavy metal and biocide genes, we calculated the ratio of non-synonymous (dN) to synonymous (dS) nucleotide substitution (dN/dS). The ratio was calculated separately for individual genes of the heavy metal operons and the biocide genes. The gene sequences were aligned using ClustalW 2.1 (Larkin et al., 2007) and the dN/dS ratios were computed using HyPhy (Kosakovsky Pond et al., 2005) implemented on the MEGA7 software (Kumar et al., 2016). Maximum likelihood was implemented as a statistical method and GTR as nucleotide substitution model. Partial deletion and site coverage cut-off value of 95% was applied to treat gaps or missing data. The overall mean distance was also calculated for each gene using MEGA7 to estimate SNP distances. Analysis was conducted using Tamura-Nei (Tamura and Nei, 1993) nucleotide substitution model with 500 bootstrap replications.

### Statistical tests

Statistical analysis was conducted using the ggstastsplot v.0.9.4 (Patil, 2021) package in R 4.1.2 (R Core Team, 2020). We used Welch’s *t*-test to compare the presence of AMR genes with the different profiles of heavy metal operon and biocide genes. We used the Pearson correlation to determine the correlation of the number of AMR gene in the genome with the number of heavy metal operons and biocide genes. We used the Pearson’s χ^2^-test to determine the association between the presence and absence of a specific AMR gene with the presence of the mercury operon. Results were considered significant when *p* < 0.05.

## Results

### Population structure of clinical *Salmonella enterica* in New Hampshire

**FIGURE 1:**
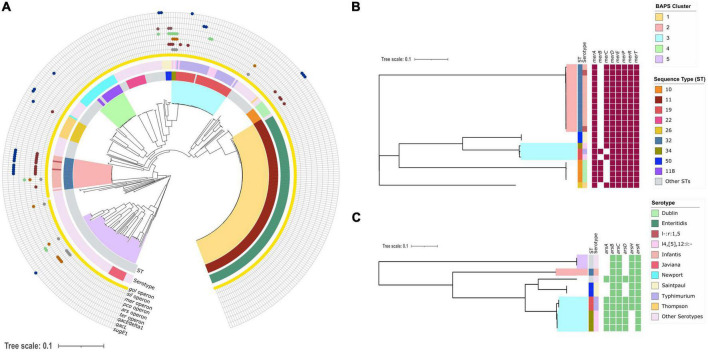
Phylogenetic distribution of heavy metal and biocide resistance genes in the New Hampshire *S. enterica* genomes. **(A)** Midpoint-rooted maximum likelihood tree showing the phylogenetic relationships of 394 *S. enterica* genomes. The tree was built using 225,784 SNPs in 3,265 core genes. Colored branches represent the five BAPS (Bayesian Analysis of Population Structure) clusters. The two inner rings represent the sequence type (ST) and serotype of each genome. The distribution of six heavy metal operons (*arsRBC*—arsenic resistance, *pcoABCDRSE*—copper resistance, *gesABC-golTSB*—gold resistance, *merRTPA*—mercury resistance, *silESRCFBA*—silver and *terBCDE*—tellurite resistance) and three biocide resistance genes (*qacEdelta1*, *qacL* and *sugE1-* quaternary ammonium compound) are shown in the outer rings. **(B)** Phylogenetic distribution of individual genes of the *mer* operon in 22 genomes. **(C)** Phylogenetic distribution of individual genes of the *ars* operon in 11 genomes. For all trees, the scale bar represents the number of nucleotide substitutions per site. Detailed information about the individual genomes is shown in Supplementary Table 1.

Our dataset consisted of 394 *S. enterica* isolates from human clinical cases in New Hampshire, USA that were collected between 2017 and 2020. The maximum likelihood phylogenetic tree generated from 3,265 core genes revealed the presence of five distinct sequence clusters (Figure 1). Overall, the population consisted of 78 STs and 67 serotypes (Figure 1A and Supplementary Table 1), with ST 11 (serotype Enteritidis) and ST 19 (serotype Typhimurium) being the most frequently detected. These two lineages consisted of 126/394 and 38/394 genomes, respectively. The first level of BAPS hierarchical clustering resulted to 129 genomes that did not group with the five largest sequence clusters. They were instead grouped in polyphyletic bins of low frequency genotypes, which BAPS has a tendency to do (Gladstone et al., 2017; Jamrozy et al., 2020).

### Prevalence of heavy metal and biocide resistance genes

We screened the genomes for the presence of heavy metal operons and biocide resistance genes using *in silico* methods (Figure 1A). We identified six different operons related to heavy metal resistance (arsenic, copper, gold, mercury, silver, and tellurite) and three genes associated with biocide resistance (*qacEdelta1*, *qacL*, and *sugE1*) in the population. The gene *sugE2* was not found in any of the genomes.

The operon conferring resistance to gold was predominant in the population, which was detected in 391/394 genomes representing 99.2% of the population (Figure 1A). The other heavy metal operons were much less common. For example, we found the *mer* operon in only 22/394 or 5.58% of the population and the *ter* operon in 3/394 or 0.76% of the population. The *pco*, *sil*, and *ars* operons were all detected in 11/394 genomes, representing 2.79% of the population. In terms of the genes associated with resistance to quaternary ammonium compounds, *qacEdelta1* and *sugE1* were detected in 15/394 and 7/394 genomes, respectively, while *qacL* was detected in only two genomes. We also detected genomes carrying between two to five heavy metal operons and biocide genes together. Only a single genome carried five operons (gold, silver, mercury, copper, and arsenic operon) belonging to ST 34 (serotype I4,[5],12:I), which was isolated in 2019. Despite the prevalence of isolates belonging to sequence cluster 1 (ST 11 serotype Enteritidis), their genomes were not associated with a high presence of heavy metal operons and biocides genes other than gold operon.

We further investigated the distribution of individual genes in the mercury and arsenic operons. The *mer* operon consists of the following genes: *merR* (mercuric resistance transcriptional regulator), *merP* (mercuric resistance system periplasmic binding protein), *merA* (mercury (II) reductase) and one or more of the mercuric transporters encoded by *merT*, merC, *merE*, *merF*, and *merG* (Nascimento et al., 2003). However, the presence of *merB* (alkylmercury lyase) and *merC* (organomercurial transporter) is not required for the full mercury resistance expression (Hamletf et al., 1992; Osborn et al., 1997; Boyd and Barkay, 2012). All 22 genomes carried the genes *merRTDPA*, but varied in the presence or absence of either *merB* or *merC* (Figure 1B). The gene *merC* was present in 17 genomes from STs 32 (serotype Infantis) and 50 (serotype Saintpaul). In contrast, the gene *merB* was present in five genomes, with four genomes from ST 10 (serotype Dublin) and one from ST 19 (serotype Typhimurium). We did not detect any genome that harbored both *merB* and *merC*.

There was also variation in gene distribution of the *ars* operon (Figure 1C). The *ars* operon consists of the following genes: *arsA* (arsenite efflux transporter ATPase subunit), *arsB* (arsenite efflux transporter ATPase subunit), *arsC* (arsenate reductase), *arsD* (arsenite efflux transporter metallochaperone), *arsH* (arsenic resistance NADPH-dependent reductase), and *arsR* (As(III)-sensing metalloregulatory transcriptional repressor) (Ben Fekih et al., 2018). Three genomes carried all six *ars* genes. There were 11 genomes that exhibited variation in the presence of the genes *arsADH*. These genomes were from STs 19 (serotype Typhimirium), 32 (serotype Infantis), 34 (serotype I4,[5],12:I), and 50 (serotype Saintpaul).

### Distribution of heavy metal and biocide genes across time, sequence types and serotypes

**FIGURE 2:**
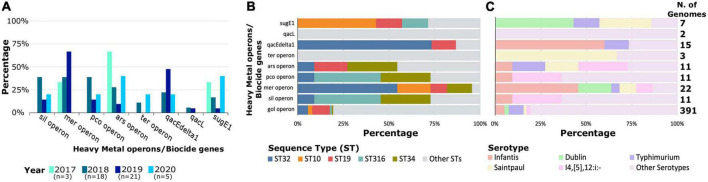
Distribution and proportion of heavy metal operons and biocide resistance genes per year **(A)**, ST **(B)**, and serotype **(C)**. The number of genomes per category is shown on the right. For visual clarity, only the most common STs and serotypes are shown. The most common ST and serotypes were those that were most frequently associated with the presence of metal and biocide operons or genes, not including the gold operon since it was present in almost all genomes. The same applies to **(A)**, the number of genomes shown below each year represents the number of genomes carrying metal and biocide operons or genes, not including the golden operon. Detailed information about the individual genomes is shown in Supplementary Table 1.

We stratified our analysis to better understand the prevalence of heavy metal operons and biocide genes in the clinical *S. enterica* population. We did not include the gold operon in our temporal analysis because it was detected in almost all isolates. First, we found that the proportion of resistance elements varied over time. The annual distribution shows that the arsenic operon and the gene *sugE1* were detected in at least one genome per year from 2017 to 2020 (Figure 2A). In 2017, three types of resistance elements were present (*mer*, *ars*, and *sugE1*) and which increased to seven in 2019 (*mer*, *sil*, *pco*, *ars*, *ter*, *qacEdelta1*, *sugE1*). We also detected a slight increase in the number of genomes carrying the mercury operon from 33% (1/3 genomes) in 2017 to 67% (14/21 genomes) in 2019.

Excluding the gold operon, which was present in 99.2% of the genomes, the other five heavy metal operons and biocide resistance elements were differentially distributed across STs (Figure 2B) and serotypes (Figure 2C). We detected 15 STs and 16 different serotypes carrying more than one resistance element. Of these, the resistance elements were commonly found in ST 32 (13/47 genomes or 27.5%) and serotype Infantis (11/47 genomes or 23.4%). The *mer* operon and *qacEdelta1* gene were detected frequently with ST 32. Of the 22 genomes carrying the *mer* operon, 12 of them were of ST 32. Similarly, of the 15 genomes carrying *qacEdelta1* gene, 11 of them were ST 32. A total of three and five types of resistance elements were found in ST 10 (serotype Dublin) and ST 19 (serotype Typhimurium/I4,[5],12:i:-), respectively. All the six heavy metal operons and three biocide resistance genes were also detected in less common STs, which may provide an important reservoir of circulating resistance elements. In terms of serotypes, six resistance elements were detected in genomes of serotype Infantis, three in serotype Dublin and five in serotype Typhimirium. Similar to STs, all nine resistance elements were detected in less common serotypes of *S. enterica.*

### Co-occurrence of heavy metal and biocide resistance genes with antimicrobial resistance genes

Heavy metals and biocides can lead to co-selection for antimicrobial resistant bacteria (Pal et al., 2015). We therefore sought to identify general patterns of co-occurrence of heavy metal, biocide and AMR genes in the *S. enterica* population. Using an *in silico* identification of AMR genes, we found a total of 58 unique genes associated with resistance to 14 antimicrobial classes (aminocoumarins, aminoglycosides, cephalosporins, cephamycins, diaminopyrimidines, fluoroquinolones, fosfomycins, macrolides, nitroimidazoles, penams, peptides, phenicols, sulfonamides, tetracyclines) (Supplementary Table 1). Of these, 16 genes were detected in all genomes (*tolC*, *sdiA*, *cpxA*, *baeR*, *acrB*, *H-NS*, *Escherichia_coli_acrA*, *CRP*, *msbA*, *mdtK*, *mdtC*, *mdtB*, *emrR*, *emrB*, *emrA*, and *bacA*). The mean number of AMR genes per genome was 25.7 (range: 21–35).

**FIGURE 3:**
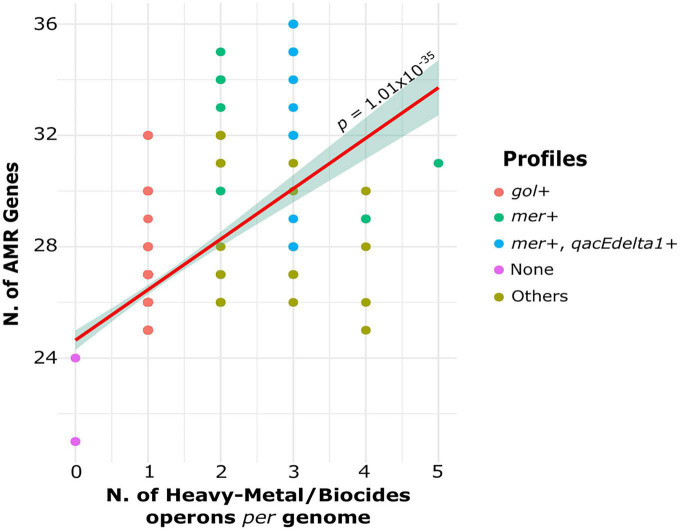
Association between the number of heavy metal operons and biocide genes per genomes with the number of AMR genes per genome. The dots are colored according to profiles determined by the presence or absence of specific resistance elements.

The number of heavy metal operons and biocide resistance genes per genome was associated with high number of AMR genes (*p* = 1.01 × 10^–35;^ Pearson’s correlation test) (Figure 3). Genomes lacking heavy metal operons and biocide resistance genes carry an average of 22 AMR genes. In contrast, genomes with 1, 2, and 3 heavy metal operons and biocide resistance genes harbor 26.4, 30.5, and 31.1 AMR genes, respectively. However, genomes with at least four heavy metal operons and biocide resistance genes carry 27.8 AMR genes.

Because the gold operon was found in almost all genomes, we also divided the genomes into different profiles to better understand the distribution of AMR genes in relation to heavy metal and biocide genes (Figure 3): *gol* + isolates carrying only the gold operon (344/394 genomes); *mer* + genomes carrying mercury operon (10/394 genomes) and other heavy metal resistance genes; m*er* + */qacEdelta1* + isolates carrying both genes (12/394 genomes). The rest of profiles were labeled as “Others” and included genomes carrying any other heavy metal operons except *mer* operon (25/394 genomes). The genomes from *mer* + and *mer* + */qacEdelta1* + profiles carried significantly more AMR genes than the genomes from the other profiles (p < 0.05; Welch’s *t*-test). The mean number of AMR genes in the *mer* + and *mer* + */qacEdelta* + profiles was 31.4 and 32.8 genes per genome, respectively. The mean number of AMR genes in genomes carrying profiles classified as Others was 28.5 genes per genome. Overall, we found an increasing trend in the number AMR genes per genome related to the presence of heavy metal operons and biocide genes in the clinical *S. enterica* population.

Next, we evaluated the association of the *mer* operon with the presence of specific AMR genes (Supplementary Table 3 and Supplementary Figure 1). Our analysis included all AMR genes found in the genomes that also carried the *mer* operon: *aac(3)-IV*, *ant(3”)-II*, *aph(3’)-Ia*, *aph(3”)-Ib*, *aph(4)-Ia, aph(6)-Id* (aminoglycoside resistance), *CTX-M-65, TEM-1, TEM-206* (cephalosporin resistance), *CMY-59* (cephamycin resistance), *dfrA14* (diaminopyrimidine resistance), *sul1 sul2* (sulfonamide resistance), and *fosA3* (fosfomycin resistance), *floR* (phenicol resistance), *tetA*, and *tetB* (tetracycline resistance). Results show that the presence of mercury operon in the genome had a strong association (*p* < 0.05; Pearson’s χ^2^-test) with the presence of the following AMR genes*: aac(3)-IV*, *ant(3”)-IIa*, *aph(3’)-Ia* and *aph(4)-Ia*, *CTX-M-65*, *dfrA14*, *sul1*, and *fosA3* (Supplementary Figure 1). Genomes harboring these AMR genes also carried the *mer* operon in frequencies ranging from 75 to 100%. In contrast, genomes not carrying these AMR genes but harbored the *mer* operon ranged in frequency between 3 and 4%.

The gene *tetA* (tetracycline resistance) was detected in 8.6% (34/394) of the *S. enterica* genomes (Supplementary Table 1), of which 53% (18/34 genomes) also carried the *mer* operon. We did not find significant association between the presence of *tetA* and the *mer* operon. However, when we examined only the genomes that are members of ST 32 (serotype Infantis/I-:r:1,5), which accounted for 64.7% (22/34) of the genomes carrying *tetA*, we observed a strong correlation between the two resistance elements (Supplementary Figure 2). Among ST 32 genomes, the *mer* operon was detected in 100% (12/12) of the genomes carrying *tetA*, while genomes that do not carry *tetA* also did not harbor the *mer* operon.

### Plasmid reconstruction and localization of resistance genes

We reconstructed the plasmids carried by the 394 *S. enterica* genomes. We identified 279 *S. enterica* genomes (Supplementary Table 1) carrying plasmids for a total of 443 plasmids (Supplementary Table 4). The plasmids ranged in size from 1,443 to 317,875 bp (mean: 55,886 bp) with the G + C content ranging from 34.07 to 61.87% (mean: 50.33%). A total of 36 different plasmid replication (Rep) proteins were identified in our dataset (Supplementary Table 4). From the 443 plasmids reconstructed, 187 had more than one rep type. IncFIB was the predominant plasmid type circulating in *S. enterica* genomes (170/443–38%). We also used the Mob-suite program to estimate the most closely related plasmid in its database and identify the taxonomy of the bacterial host. The plasmids circulating in our dataset were most closely related to plasmids present in 22 different species, including *Klebsiella pneumoniae*, *Shigella flexineri*, and *Escherichia coli*.

**FIGURE 4:**
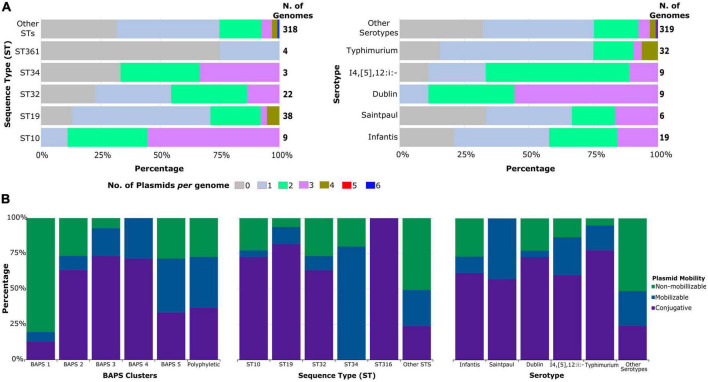
Plasmid content of *S. enterica* genomes. **(A)** Proportion of the number of plasmids per genome classified according to ST and serotypes. **(B)** Distribution of plasmid mobility types according to sequence clusters, STs, and serotypes. For visual clarity, only the most common STs and serotypes are shown, while less common STs and serotypes were grouped together. Detailed information about the individual genomes is shown in Supplementary Table 4.

We detected the presence of 0–6 plasmids present per genome (mean: 1.43 plasmids per genome). Only two genomes both from 2019 carried six plasmids each, with one belonging to ST 22 (serotype I-:e,h:e,n,z15) and another to ST 33 (serotype Hadar). Among the ST 32 (serotype Infantis/I-:r:1,5) genomes, a total of 31.8% (7/22) carried one or two plasmids (Figure 4A). More than half of the genomes from ST 10 (serotype Dublin) carried three plasmids per genome (55.5% or 5/9 genomes). For those genomes from serotype I4,[5],12:i:- (ST 19/ST 34), 55.5% (5/9 genomes) carried two plasmids.

Our results also revealed that 42.2% or 187/443 of the predicted plasmids were non-mobilizable (Supplementary Table 4). A total of 35.2% (156/443 plasmids) were conjugative, while 22.6% (100/443) were mobilizable. Apart from ST 34, the most frequent STs carrying heavy metal/biocide resistance elements were enriched with conjugative plasmids (Figure 4B). The presence of conjugative plasmids in those STs ranged from 63% (19/30 plasmids) in ST 32 genomes to 81% (40/49 plasmids) in ST 19 genomes. The single plasmid found in genomes from ST 316 was also conjugative. Genomes from ST 34 were the only ones that carry more mobilizable plasmids (80% or 4/5 plasmids) than others. Conjugative plasmids were also predominant in the most frequent serotypes associated with heavy metal/biocide resistance. The presence of non-mobilizable plasmids was more enriched in less common STs and serotypes.

We re-annotated both plasmids and chromosomes to determine if the heavy metal operons, biocides resistance genes and AMR genes were located on either chromosome or plasmids (Table 1 and Supplementary Table 1). The *gol* operon was located on the chromosome. Majority of isolates carrying the *mer* operon, *ter* operon, *qacEdelta1*, *qacL*, and *sugE1* carried these resistance elements in plasmids. The operons related to silver and copper resistance (*sil* and *pco*) were exclusively located within the chromosome. Genomes harboring the *ars* operon often had the element present within the chromosome (*n* = 9); however, two isolates carried the operon on conjugative plasmids. The plasmids responsible for carrying heavy metal operons and biocides genes were predominantly conjugative type. The IncFIB plasmid was the predominant plasmid type for carrying the *mer* operon and *qacEdelta1*, while IncI-gamma was the predominant type in genomes harboring *qacL* and *sugE1*. The three genomes carrying *ter* operon and the only two genomes carrying the *ars* operon carried them in IncHI2A/rep_cluster_1088 plasmids.

**TABLE 1:** Distribution of heavy metal operons and biocides genes according to genetic localization, plasmid mobility, and plasmid type.

Heavy metal operon	Localization		Plasmid mobility	Plasmid type
	Chromosome (*n*)	Plasmid (*n*)		
*gol* operon	392	0	−	−
*sil* operon	11	0	−	−
*mer* operon	1	21	Conjugative (20) Non-mobilizable (1)	IncFIB(11) IncI-gamma(3) IncQ1(2) IncC(2) IncFII(1) IncX1 (1) IncHI2A(2) Rep_cluster_1088 (2)
*pco* operon	11	0	−	−
*ars* operon	9	2	Conjugative (2)	IncHI2A(2) Rep_cluster_1088 (2)
*ter* operon	0	3	Conjugative (3)	IncHI2A(3) Rep_cluster_1088 (3)
**Biocide genes**				
*qacEdelta1*	2	13	Conjugative (12) Mobilizable (1)	IncFIB(11) IncI-gamma(1) IncR(1)
*qacL*	0	2	Conjugative (2)	IncI-gamma(2)
*sugE1*	0	7	Conjugative (7)	IncI-gamma(3) IncC(4)

n, number of isolates.

Finally, our plasmid analysis confirmed the co-occurrence of AMR genes and heavy metal operons and biocide resistance genes, corroborating previous results showing correlation between the *mer* operon and genes related to aminoglycoside resistance, cephalosporin, diaminopyrimidine, sulfonamide, and fosfomycin resistance (Supplementary Table 1). Plasmids carrying mercury operon also co-carried several of the genes associated with resistance to those antimicrobial classes and *qacEdelta1* gene. Gene content of different plasmid types was also variable, with plasmids carrying the *mer* operon also harboring between one to ten AMR genes.

### Heavy metal operons and biocide resistance genes are under negative selection

**FIGURE 5:**
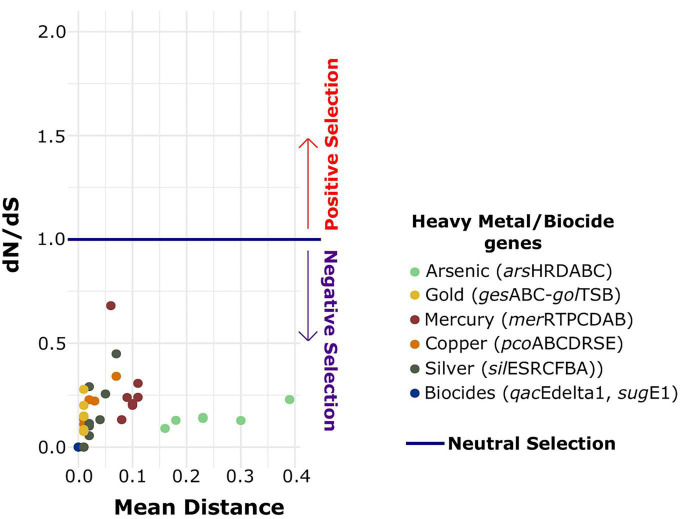
Association between mean pairwise SNP distances and dN/dS value for individual genes of each heavy metal operon and biocide resistance genes. The dots are colored according to the identity of each resistance element.

We estimated the strength of selection pressure on the heavy metal and biocide resistance genes by calculating the ratio of non-synonymous to synonymous polymorphisms (dN/dS) for individual genes. All genes were under negative or purifying selection, with dN/dS values ranging from 0.00 to 0.68 (Figure 5). The dN/dS values ranged from 0.09 (*arsD*) to 0.22 (*arsR*) for *ars* operon and from 0.07 (*gesB*) to 0.27 (*gesA*) for *gol* operon. The genes from *mer* operon had the greatest variation in the dN/dS values, ranging from 0.13 (*merT*) to 0.68 (*merC*). For *pco* operon, dN/dS values ranged from 0.0 (*pcoC*) to 0.34 (*pcoE*), while for *sil* operon dN/dS values ranged from 0.0 (*silR*) to 0.44 (*silA*). The dN/dS values estimated for *qacEdelta1* and *sugE1* were 0.0 for both genes. Lastly, the overall mean of all individual pairwise distance among the nucleotide sequences for each gene ranged from 0.0 to 0.39. The genes of the arsenic operon were the most divergent gene sequences observed.

## Discussion

There is growing concern that the widespread presence of heavy metals and biocides in the environment to which bacterial pathogens are directly and indirectly exposed to may act as strong selective agents that contribute to the proliferation and persistence of AMR. In our study, we characterized the presence of genes related to heavy metal and biocide resistance in *S. enterica* population from clinical human-derived specimens in New Hampshire, USA from 2017 to 2020. Our results demonstrate the presence of different operons associated with arsenic, copper, gold, mercury, silver, and tellurite resistance. We also detected three genes related to quaternary ammonium compound resistance, a type of chemical biocide routinely used in household and industrial products (Zhang et al., 2015). These resistance operons and genes were differentially distributed across multiple STs and serotypes. These genes also experience strong negative selection, with some of these genes frequently co-occurring with AMR genes within the same plasmid type. The presence of the *mer* operon and the *qacEdelta1* gene was positively correlated with an increase in the number of AMR genes. Recently, a study analyzing 56,348 *Salmonella* genomes publicly available also confirmed the co-occurrence of mercury and beta-lactams, sulfonamides and tetracyclines (Fenske and Scaria, 2021). Heavy metals, biocides and other toxic compounds can therefore act as agents of co-selection and co-dissemination with AMR genes (Vats et al., 2022).

Except for the gold operon which was detected in nearly all genomes, the other five heavy metal operons were present at low frequencies. The percentage of genomes harboring the *mer* operon in our population was 5.58%. In contrast, it is present in approximately 20% in food-derived *S. enterica* (Deng et al., 2018; Yang et al., 2020). The presence of silver and copper resistance has been also described in high frequencies in food- and animal-derived *S. enterica* isolates (Mourão et al., 2016; Ferreira et al., 2019; Yang et al., 2020). The differential frequencies of heavy metal operons could be due to different selection pressures that exist in different settings. Moreover, the success of a particular gene could also be explained by the genetic background in which the gene is carried in. Both the *sil* and *pco* operons have been frequently found in *S. enterica* isolates and are co-located on the IncHI2 plasmid, one of the most successful plasmids in MDR *Salmonella* species (García-Fernández and Carattoli, 2010; Hoffmann et al., 2014; Chen et al., 2016; Zhao et al., 2018; Ferreira et al., 2019). All the isolates in our study harboring the *sil* operon also carried the *pco* operon. However, genomes from our data set carried both genes within the chromosome.

Beyond the presence in plasmids, *sil* and *pco* operons have been described in the genomic island SGI-4 (Petrovska et al., 2016). The presence of SGI-4 has been strongly associated to the emergence of the serotype 4,[5],12:i:-/ST34, an important *S. enterica* lineage. This lineage has been considered a pandemic clone associated with livestock and related to many human outbreaks around the world (Hauser et al., 2010; Hopkins et al., 2010; Mulvey et al., 2013; Mourão et al., 2015). The SGI-4 genomic island was identified in over 95% of the 797 clinical isolates belonging to this lineage in England and Wales (Branchu et al., 2019). Serotype 4,[5],12,i:-/ST34 is a monophasic *S. typhimurium* variant incapable of producing the second-phase flagellar antigen (Moreno Switt et al., 2009). In our study, three genomes belonging to serotype 4,[5],12:i:-/ST34 carried the *pco* and *sil* operons. We also identified six serotypes and five STs carrying those elements within the chromosome, with the ST 316 (Serotype Montevideo/I-:g,m,s:-) being predominant. Although we did not confirm whether the *pco* and *sil* operons were present in the genomic island SGI-4, our data raise the importance of other mobile genetic elements in disseminating heavy metal and biocide resistance genes in different *S. enterica* populations.

Recently, a study conducted in monophasic isolates from Canada also found the arsenic resistance element (*ars* operon) and the presence of a discrete chromosomal island associated to mercury resistance (MREL—mercury resistance element) inside the genomic island SGI-4 (Clark et al., 2020). These elements have been usually found inserted near by the *iroB* locus leading to disruptions in the second phase antigen region (Clark et al., 2020). In the *S. enterica* genomes from New Hampshire, *ars* operon was found within the chromosome, while the *mer* operon was frequently carried in plasmids. The *mer* operon is usually related to the Tn*21*, a successful transposon involved in the global dissemination of AMR determinants in gram-negative bacteria (Essa et al., 2003; Guerra et al., 2004; Skurnik et al., 2010). The Tn*21* family usually presents a class I integron encoding a site-specific system for the acquisition of multiple AMR genes, genes for its own transposition, and the *mer* operon (Liebert et al., 1999). Integrative and conjugative elements (ICEs) are usually prone to many rearrangements. The single isolate carrying *mer* operon inserted in the chromosome as well as the two isolates carrying the *ars* operon in plasmids may reflect their mobility between the chromosome and plasmids. Moreover, the differential distribution of *mer* and *ars* genes across very closely related genomes we found in our study could also be consequence of either a true gene loss caused by their frequent movement or errors due to sequencing and assembly.

Our results also highlight the wide distribution of gold operon in clinical human isolates. A recent study reported the presence of the gold operon in all 375 *S. enterica* isolates from wild birds in the United States (Fu et al., 2021). The widespread distribution of the gold operon in *S. enterica* isolates from clinical and environmental sources may suggest the importance of this operon to the survival of the species. The gold operon has been proposed to be important not only for gold tolerance but for copper homeostasis in *Salmonella* since this bacterium does not have the *cus* operon (related to copper homeostasis) (Osman and Cavet, 2011; Méndez et al., 2022). All heavy metal and biocide resistance genes in our study were under purifying selection, whereby genetic variants that are detrimental are removed. Our results suggest that these genes are under pressure to stay the same because they are critical for the organism to function correctly, which may explain in part their success to thrive in different environments.

We acknowledge several limitations of our study. Sampling was limited to only clinical specimens received by DHHS. Hence, we were not able to precisely determine the heavy metal and biocide resistance profiles of *S. enterica* from animals, food products and the environment in New Hampshire. It is possible that *S. enterica* from non-human isolates may exhibit different phylogenetic distribution of these genes and association with AMR genes. Moreover, because we had different number of isolates for each year of sampling, it is unclear if the increase in the prevalence of some genes is due to sampling bias. Future work should implement a more systematic and broad sampling of *S. enterica* from multiple settings, especially at the local level. Moreover, our analyses were based on *in silico* determination of heavy metal and biocide resistance genes; hence, we recognize that different resistance gene detection programs have unique strengths and weaknesses (Davies et al., 2021). Development of improved genotyping/AMR detection approaches are therefore needed to distinguish closely related gene variants and to precisely determine true gene loss caused by their frequent movement. Additionally, we cannot confirm if these operons and genes are functional, which can influence their potential to co-select other genetic elements. Future work should focus on phenotypic tests of heavy metal and biocide resistance.

In summary, our analysis revealed valuable insights about the presence and distribution of heavy metal and biocide resistance genes in human-derived *S. enterica* population and their potential impacts for co-selection and mobility with AMR genes. Our study highlights the need for continued surveillance of STs and serotypes that frequently carry those genes that confer resistance to heavy metals, biocides, disinfectants and other toxic compounds.

## Data availability statement

The datasets presented in this study can be found in online repositories. The names of the repository/repositories and accession number(s) can be found only in the Supplementary material.

## Ethics statement

All *S. enterica* sampling and whole genome sequencing were carried out for disease surveillance purposes as part of the CDC PulseNet program. Samples used in the study were subcultured bacterial isolates that had been archived in the routine course of clinical laboratory operations. The New Hampshire Public Health Laboratories protocol for bacterial disease surveillance utilizing whole genome sequencing does not include human subjects research and hence, no review or approval from Institutional Review Board (IRB) was needed. No patient specimens were used and patient protected health information was not collected. Therefore, informed consent was not required.

## Author contributions

SS and CPA designed the work and wrote the manuscript with contributions from all authors. SS and MT carried out all bioinformatics analyses. JL, XZ, KW, FG, and CB carried out the data collection and laboratory work. CPA guided the work. All authors read, edited, and approved the final manuscript.

## Footnotes

1
https://www.cdc.gov/pulsenet/pdf/P/NL38-WGS-on-MiSeq-508.pdf

2
https://github.com/tseemann/shovill

3
https://github.com/tseemann/mlst

4
https://github.com/tseemannabricate

## Supplementary material

Supplementary Figure 1 Association of the *mer* operon with the presence of specific AMR genes. This figure only shows those genes where statistically significant correlation was detected. Detailed information for all the remaining genes analyzed but where statistically significant correlation was not found shown in Supplementary Table 3.

Supplementary Figure 2 Association of the *mer* operon with the *tetA* gene for the entire dataset **(left)** and ST 32 genomes only **(right)**.
