# Supplementary figures and images for: Population analysis of heavy metal and biocide resistance genes in Salmonella enterica from human clinical cases in New Hampshire, United States
Source: Front Microbiol. 2022 Oct 19;13:983083. doi: 10.3389/fmicb.2022.983083 (PMC9626534; doi:10.3389/fmicb.2022.983083)

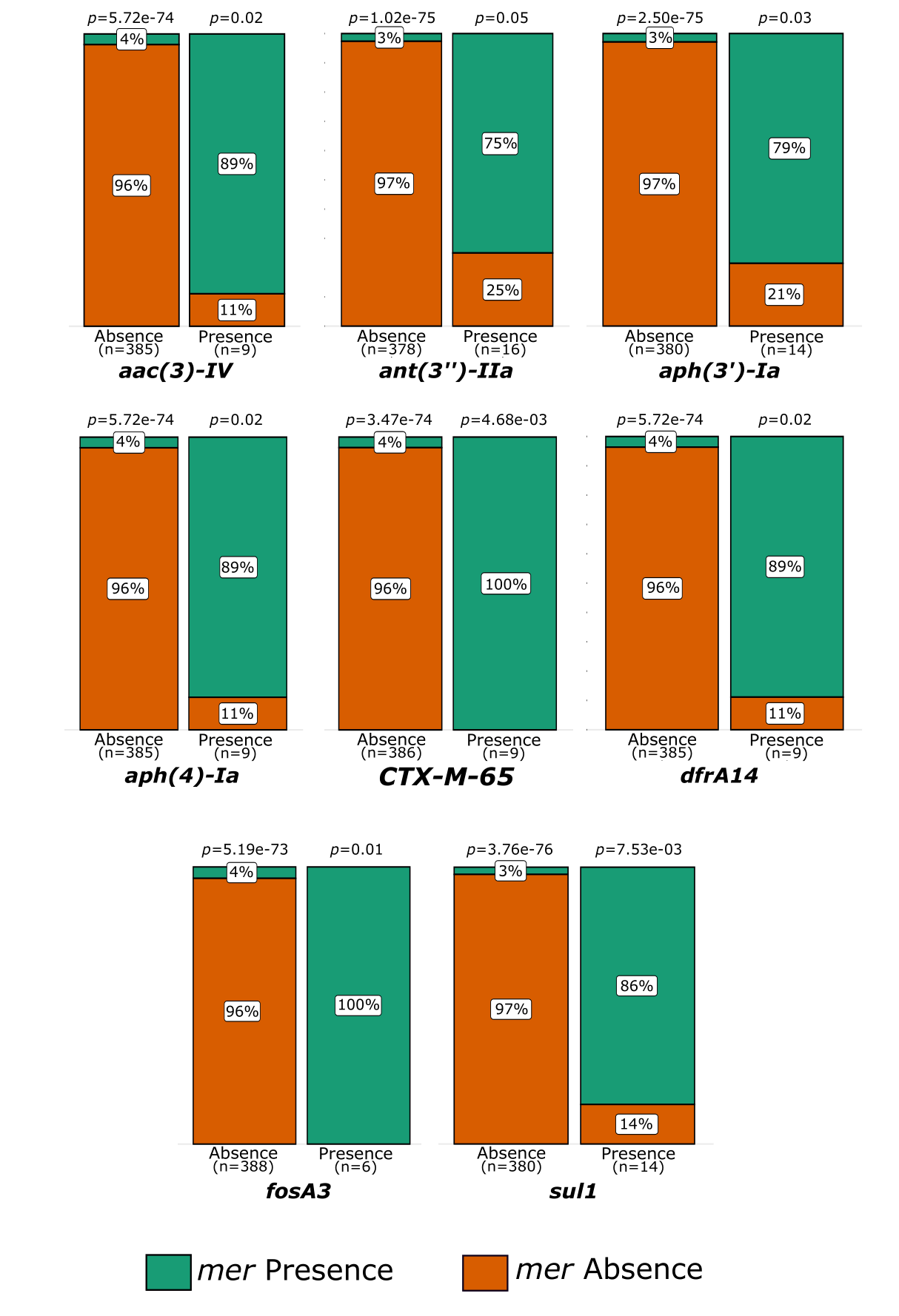

Supplement: Supplementary Figure 1 — Association of the mer operon with the presence of specific AMR genes. This figure only shows those genes where statistically significant correlation was detected. Detailed information for all the remaining genes analyzed but where statistically significant correlation was not found shown in Supplementary Table 3. [file Image_1.TIFF]

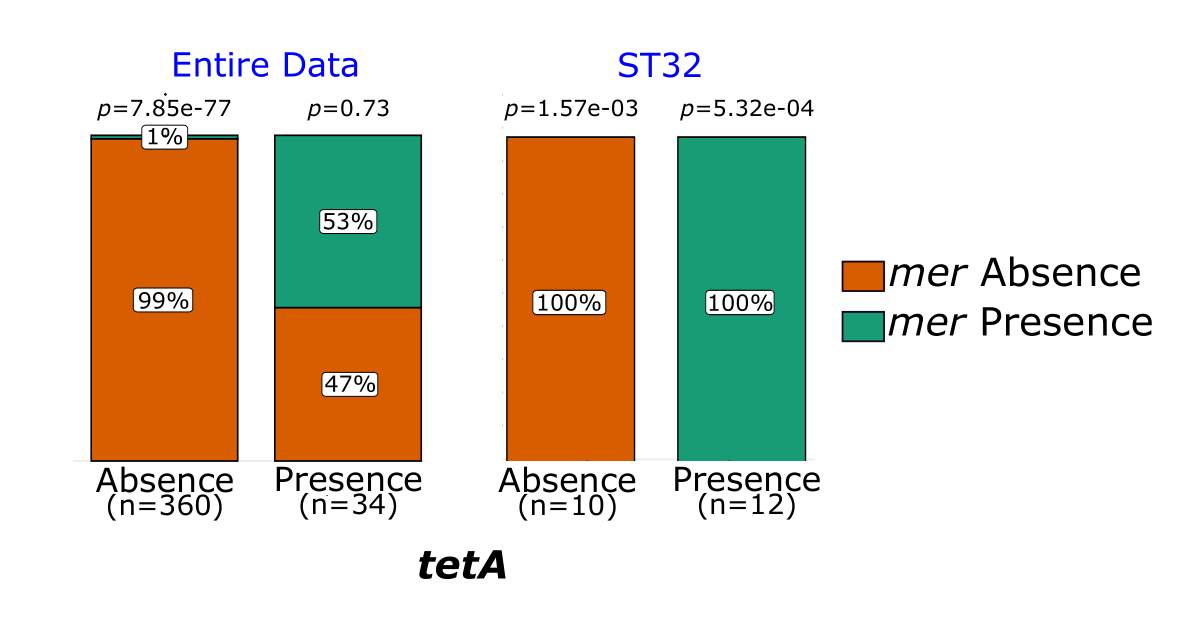

Supplement: Supplementary Figure 2 — Association of the mer operon with the tetA gene for the entire dataset (left) and ST 32 genomes only (right). [file Image_2.TIFF]
